# Keystone interdependence: Sea otter responses to a prey surplus following the collapse of a rocky intertidal predator

**DOI:** 10.1126/sciadv.adu1028

**Published:** 2025-04-30

**Authors:** Joshua G. Smith, Jessica A. Fujii, Rani Gaddam, Leilani Konrad, Sophia Lyon, Teri E. Nicholson, Peter T. Raimondi, April D. Ridlon, Michelle Staedler, Joseph A. Tomoleoni, Julie L. Yee, M. Tim Tinker

**Affiliations:** ^1^ Conservation and Science Division, Monterey Bay Aquarium, Monterey, CA, USA.; ^2^ Department of Ecology and Evolutionary Biology, University of California, Santa Cruz, CA, USA.; ^3^ U.S. Geological Survey, Western Ecological Research Center, Santa Cruz, CA, USA.; ^4^ Nhydra Ecological Consulting, Head of St Margarets Bay, Nova Scotia, Canada.

## Abstract

The sea star *Pisaster ochraceus* and sea otters (*Enhydra lutris*) are two predators capable of shaping rocky intertidal and kelp forest community structure and functioning. In 2013, a sea star wasting event decimated populations of *Pisaster* along the west coast of North America. The collapse of this species in the rocky intertidal revealed an unexpected relationship between two keystone predators. In this study, we show how the loss of *Pisaster* along the Monterey Peninsula, CA, USA led to an increase in mussel (*Mytilus californianus*) size and expansion into lower tidal zones. Before the sea star wasting event, the local sea otter population fluctuated around a near equilibrium. However, in the absence of *Pisaster*, sea otters increased their dietary intake on mussels, which contributed in part to a local population-level rise. These results demonstrate how the loss of a keystone predator in one ecosystem may impart population-level changes to another.

## Teaser

Keystone interdependence occurs when shifts in one keystone species affect another, linking ecosystems across boundaries.

## INTRODUCTION

Understanding mechanisms of community regulation remains a central goal in ecology. Ecologists have long recognized that collections of populations relate through positive and negative interactions, often within spatially discrete habitats (*1*). Interactions may also occur across habitats and ecosystem boundaries, linking energy transfer and thus coupling functioning via the movement of resources (*2*–*5*). Although transboundary movement of resources between ecosystems (e.g., migration, nutrient cycling, connectivity through dispersal, etc.) is common, it remains unclear how changes in community regulation within discrete habitats may impart cross-ecosystem coupling.

Some of the most prominent efforts to understand community regulation are centered around identifying the environmental and ecological contexts that shape the relative contribution of bottom-up and top-down forcing (*6*–*8*). Energy transfer from primary producers to consumers can drive the control of communities and ecosystems, but higher-level predators can determine the distribution of energy flow throughout systems (*6*). Both of these processes can ultimately influence community structure, functioning, and persistence over space and time (*9*, *10*). However, in systems under relatively strong top-down control, environmental or biological perturbations may markedly alter community regulation (*7*, *11*). Events (e.g., disease-driven mass mortality, herbivore outbreaks, severe storms, heatwaves, etc.) that shift the strength of community regulation may reveal energetic pathways and the processes that facilitate functioning within and across ecosystems (*12*, *13*).

The rocky intertidal and kelp forests are two ecosystems often found adjacent to one another in temperate coastlines around the world. In both ecosystems, macroalgae and plankton are the two primary sources of productivity (*14*). In the rocky intertidal, the proliferation of planktivorous mussels can alter community composition (*15*). However, predation on mussels by mesopredators, such as the sea star *Pisaster ochraceus* (hereafter *Pisaster*), is known to exert strong top-down effects (*16*, *17*). *Pisaster* is recognized as a keystone predator in the rocky intertidal because of its ability to regulate the spatial distribution of mussels (*Mytilus californianus)* (*16*). Similarly, in kelp forests, sea otters (*Enhydra lutris*) can suppress herbivore abundance, thereby having positive indirect benefits on macroalgae production and abundance (*18*). In areas where *Pisaster* and sea otters are highly abundant, prey limitation can increase biodiversity, drive dietary specialization [in sea otters, (*19*)] and stabilize population dynamics and predator-prey interactions (*20*).

Given the disproportionate effect that keystone predators exert on their prey populations, declines in the abundance of a keystone predator in one ecosystem may result in sudden prey increases that could subsidize predator populations in adjacent or connected ecosystems. While there is some evidence for changes in keystone predator populations in marine systems altering community regulation in terrestrial systems (*3*, *21*), cross-ecosystem coupling between coastal and marine ecosystems is not widely documented [but see (*21*) for an island system]. Therefore, understanding how predation couples ecosystems is essential to fully capturing the within- and cross-ecosystem impacts of keystone species.

Along the central coast of California, USA, populations of the southern sea otter (*E. lutris nereis*) increased beginning in the late 1950s, following their near extirpation during the fur trade (*22*). The local subpopulation (i.e., Monterey to Big Sur, California) stabilized around the late 1980s and fluctuated around an equilibrium for the next three decades (*22*, *23*). During this period of slow population growth, sea otters consumed a diverse diet of marine invertebrates and developed individual diet specializations on subsets of invertebrate prey to reduce intraspecific competition (*19*). However, beginning in 2013, a coastwide sea star epizootic decimated populations of *Pisaster*, among other species (*24*). The near extirpation of a once highly abundant keystone mesopredator presented an opportunity to explore how the predatory release of prey (mussels) in the rocky intertidal may have resulted in population-level changes to a kelp forest-associated predator (sea otters) and to evaluate how this diet shift imparts cross-ecosystem consequences.

Here, we examine how environmental and ecological conditions influence coupling between the rocky intertidal and kelp forests and the relative contribution of top-down versus bottom-up forcing on community regulation. We synthesize two decades of sea otter foraging data with long-term rocky intertidal monitoring surveys to test the hypotheses that: (i) The sudden collapse of the keystone predator *Pisaster* in the rocky intertidal zone led to an expansion of mussel populations into lower tidal zones. (ii) The increased abundance and lower distribution of mussels enhanced their accessibility to sea otters, resulting in a measurable increase in mussel consumption reflected in sea otter dietary intake. (iii) The elevated energetic intake from mussels contributed to a population-level increase in sea otters beyond their prior decadal equilibrium. (iv) The shift in sea otter diet toward increased consumption of intertidal mussels signifies that cross-ecosystem coupling can occur through the loss of a keystone predator and that these changes impart population- and ecosystem-level consequences.

## RESULTS

**Fig. 1.**
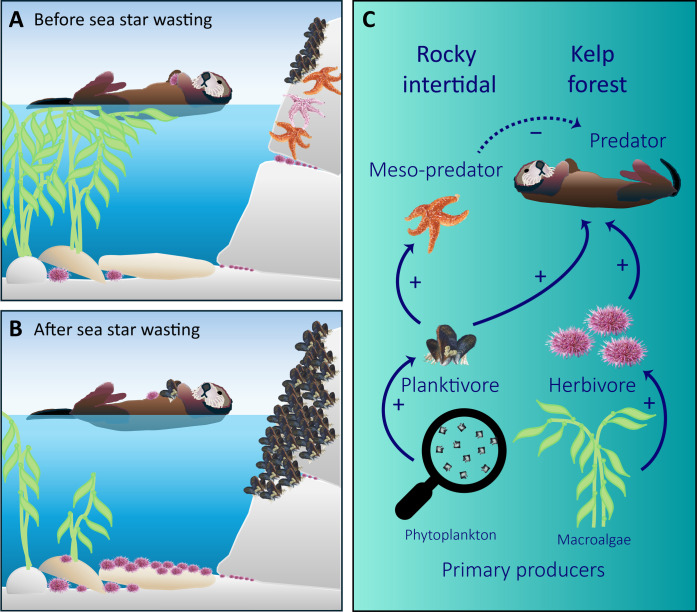
Increases in mussel cover associated with the loss of an intertidal mesopredator reveal mechanisms of ecosystem coupling through indirect trophic interactions. In (**A**), the lower distribution of mussels (*M. californianus*) is limited by predation from the sea star *P. ochraceus* and sea otters primarily forage on subtidal invertebrates. In (**B**), mussels expand into the lower rocky intertidal following a coast-wide sea star wasting event that decimated populations of *Pisaster*. In the absence of *Pisaster*, mussels increased in cover and expanded lower within the intertidal zone, enhancing their availability to foraging sea otters and serving as a prey surplus. In (**C**), focal energetic pathways for rocky intertidal and kelp forest ecosystems are depicted with a series of arrows. Solid arrows indicate direct relationships, and the dotted arrow indicates indirect keystone interdependence between *Pisaster* and sea otters. J.G.S. conceptualized the figure, and T.E.N. created the figure. Illustration © Monterey Bay Aquarium.

**Fig. 2.**
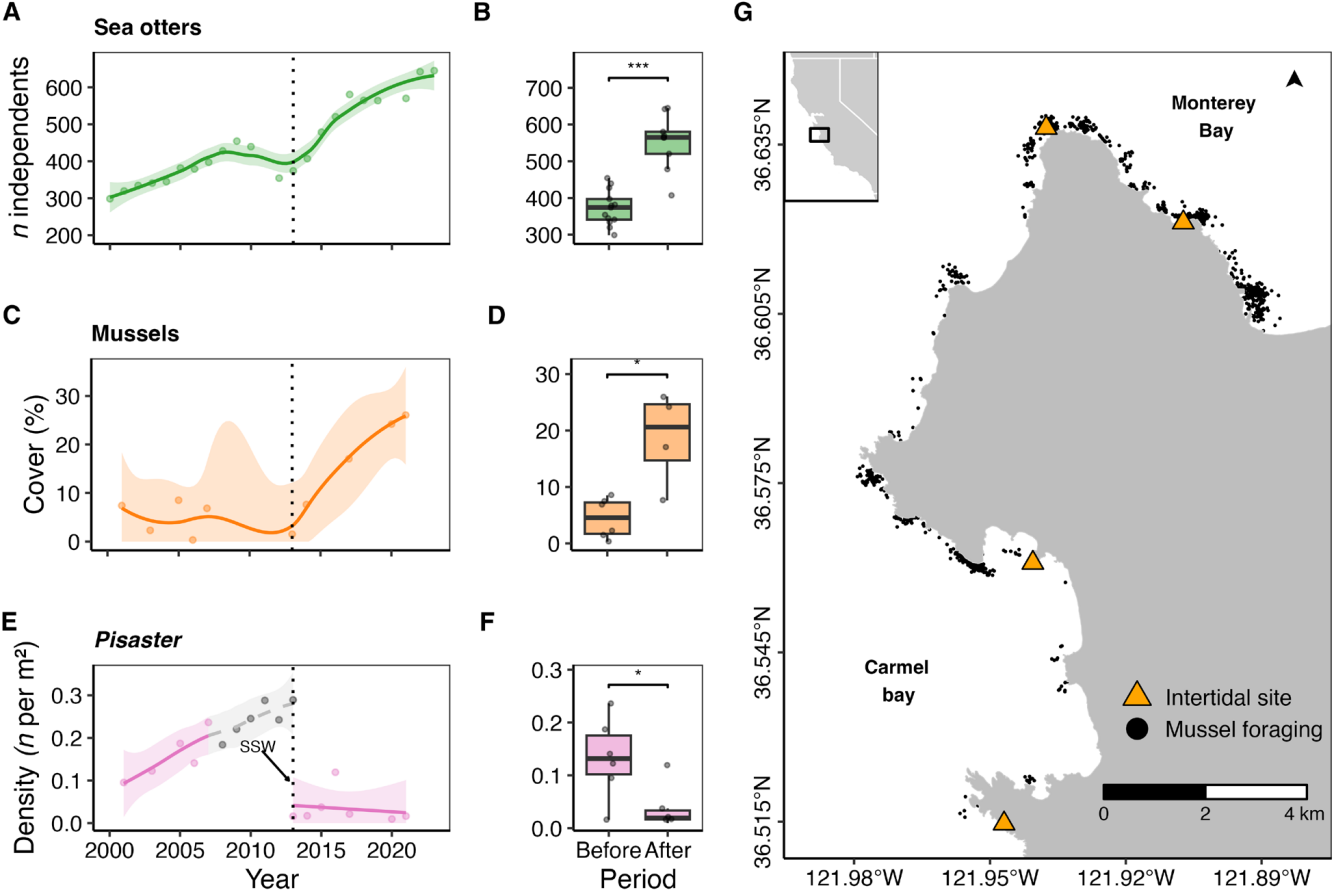
Temporal changes in the abundance of sea otters, mussels, and *Pisaster* along the central coast of California, USA. In (**A**), total counts of sea otters observed throughout the study area are shown as points for each year fit with a cubic spline and standard error ribbon. Panel (**B**) depicts a boxplot with counts before versus after the 2013 sea star wasting (SSW) event. In (**C**), mean percent cover of mussels are shown for long-term rocky intertidal monitoring sites fit with a cubic spline and a standard error ribbon. Panel (**D**) depicts a boxplot with percent cover before vs. after SSW. In (**E**), the mean density of *P. ochraceus* is shown for each year. Years before 2008 were fit using Gompertz growth and extrapolated through 2013 (dashed gray line and gray points; data were not collected during the 2008–2012 period). Panel (**F**) depicts boxplot with *Pisaster* density before versus after SSW. The timing of the 2013 sea star wasting event is shown as the vertical dotted line through (A), (C), and (E). Boxplot brackets and asterisks denote significance between periods (*P < 0.05 and ***P < 0.001) as determined by a *t* test. Panel (**G**) depicts the study area with sea otter forage bout locations for mussels (black dots) and rocky intertidal long-term monitoring sites (orange triangles).

We found evidence of keystone interdependence and cross-ecosystem coupling initiated by the loss of *P. ochraceus* in the rocky intertidal (Figs. 1 and 2). The collapse of this keystone mesopredator was temporally correlated with an observed increase in mussel (*M. californianus*) cover (Fig. 2, C to F). An abrupt decline in *Pisaster* density was first observed in 2013, and mussel cover increased beginning around 2015. A slight increase in sea otter abundance began in 2014 and continued with increasing mussel cover (Fig. 2, A to D).

### Predatory release and expansion of mussels in the rocky intertidal

**Fig. 3.**
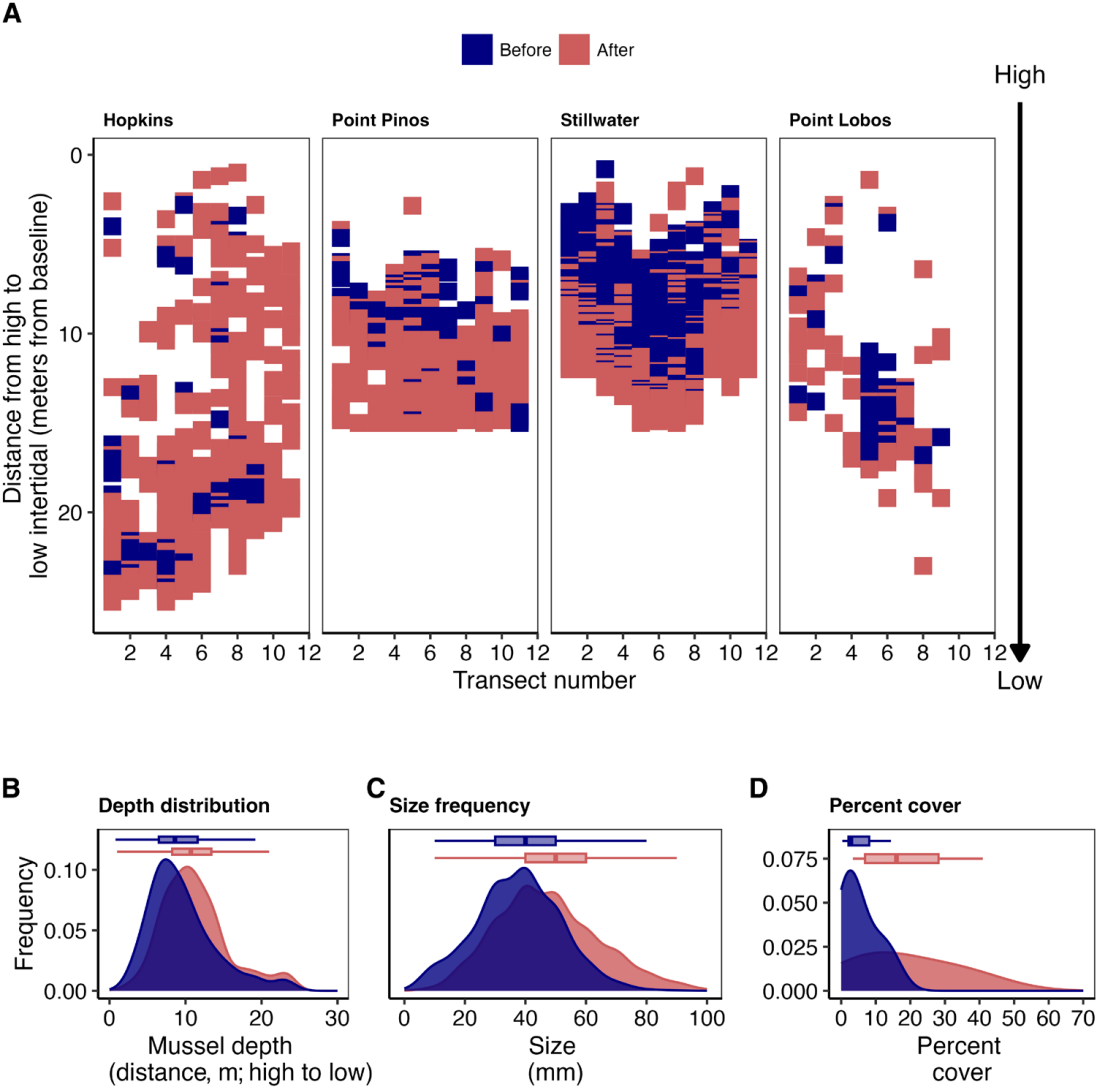
Mussel expansion from the high to lower rocky intertidal at four long term monitoring sites. Panel (**A**) depicts the presence of mussels at replicate transects (*x* axis) along a gradient from high to low (*y* axis) in the rocky intertidal. Increasing distance represents a lower position in the rocky intertidal. Mussel presence is shown in blue for all years sampled pre-sea star wasting, and the post-sea star wasting period is shown in red, indicating the presence of mussels at locations where they were not observed before the sea star wasting event. In (**B**), frequency distribution of mussel presence from high to the low intertidal, (**C**) size frequency distribution of mussels, and (**D**) frequency of mussel percent cover.

Following the 2013 sea star wasting event, mussel distribution expanded throughout the rocky intertidal (Fig. 3). By comparing the relative distribution of mussels at four sites before (2009 to 2012) versus after (2013 to 2021) the sea star wasting event, we found that mussels increased throughout the rocky intertidal and also expanded from the upper intertidal toward the mean lower low water (MLLW) line (Fig. 3A). Across the four sampled sites, the distribution of mussels shifted between the two time periods toward an overall mean lower low water distribution (9.59 ± 4.60 to 12.1 ± 4.71 m) after the decline in *Pisaster* (*P* < 0.001, *t* = −11.281, df = 1469.6; Fig. 3B). The mean size of mussels also significantly increased (from 37.5 ± 13.8 to 47.6 ± 16.9 mm; *P* < 0.001, *t* = −16.445, df = 2343 Fig. 3C), especially for the upper quartile. Correspondingly, mean mussel cover expanded significantly from 5.36 to 18.4% (*P* < 0.05, *t* = −2.771, df = 12.022; Fig. 3D) within sampled intertidal areas during this same time period.

### Sea otter dietary composition and energetic intake

**Fig. 4.**
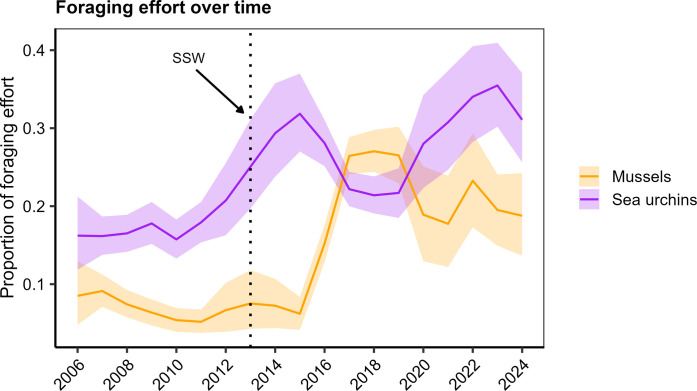
Proportion of sea otter foraging effort allocated to mussels (orange) or sea urchins (purple) over time. Foraging effort reflects the proportion of total feeding activity devoted to dives targeting primarily urchins or primarily mussels, averaged across all included foraging observations and corrected for observation and sampling biases. Error ribbons are shown as 95% credible intervals. Last, the vertical dotted line represents the onset of the 2013 sea star wasting (SSW) event.

Annual variation in sea otter dietary composition and caloric intake reflected cross-ecosystem level changes in prey availability (Fig. 4). Our analyses revealed that before 2013, the relative proportion of foraging effort for sea urchins was higher than mussels (17.2 ∓ 0.6% versus 6.9 ∓ 0.4%, respectively). However, the foraging effort on sea urchin prey increased coinciding with the 2014 outbreak of sea urchins in the subtidal (Fig. 4). This is likely reflective of a sudden shift in the behavior of sea urchins that made them more conspicuous, as a result of declines in kelp availability (*25*). The proportion of sea otter foraging effort on mussels was relatively low (<7% ± 0.5 SE) before the sea star wasting event but increased 2.5-fold to 17.7 ± 2.1% in the post-2013 period (Fig. 4 and fig. S2). The initial increase in mussel consumption began in 2016 after an apparent 3-year lag following the 2013 sea star wasting event.

**Fig. 5.**
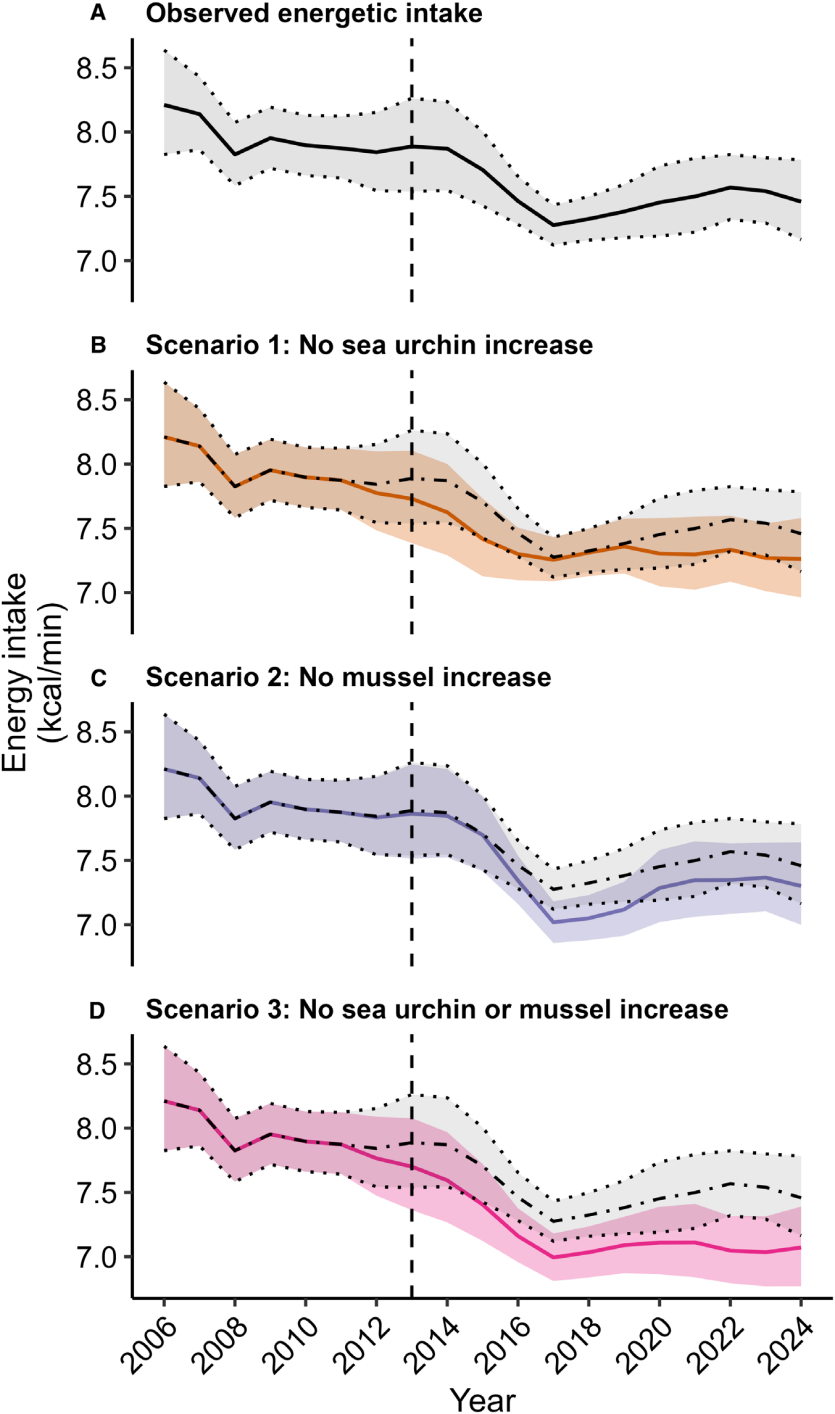
Trends in sea otter energy intake rates while foraging, as estimated from data collected in field surveys and analyzed using a Bayesian hierarchical model. Panel (**A**) shows trends reflecting observed prey population dynamics. Lower panels (**B** to **D**) compare observed trends (light gaey shaded band) to predicted trends under three alternative scenarios: (B) no post-2012 increase in urchin density, (C) no post-2012 increase in mussel density, and (D) no post-2012 increase in urchins or mussels. For each plot, the shaded bands indicate 95% credible intervals, and solid lines indicate point estimates of energy intake rates.

A Bayesian model of sea otter prey patch selection and bioenergetics revealed how shifts in the relative density of different prey taxa, combined with sea otters tendency to allocate foraging effort to maximize expected energy intake, determined trends in energy gain and prey consumption rates. The increase in sea urchin abundance after 2013 was accompanied by an apparent decline in encounter rates with more energetically profitable and preferred prey types (including abalone, Cancrid crabs, kelp crabs, sea stars and cephalopods; fig. S2), perhaps caused by a reduction in kelp habitat available for these species (*26*). These trends combined to drive an increase in foraging effort allocated to less energetically profitable urchins (Fig. 4) and an associated increase in urchin consumption rates (fig. S1). By 2016, increased density of mussels, combined with fluctuations in the density of suitable urchin prey patches, resulted in an increase in foraging effort allocated to mussels as well as urchins (Fig. 4). In general, the reduced prevalence of more profitable prey types and associated shift toward a diet dominated by sea urchins and mussels caused a net reduction in the overall rate of energy gain after 2013 (Fig. 5A).

By resolving our model under alternative prey dynamics, we inferred expected trends in energy intake under scenarios in which increases in sea urchins and mussels did not occur. The initial surge in sea urchin abundance in 2014–2015 appeared to allow for a notable uptick in energy intake relative to a scenario with no sea urchin increase (Fig. 5B). Similarly, increased mussel abundance contributed to higher than expected energy intake rates between 2016 and 2019 (Fig. 5C). The combined increases of both species allowed sea otters to sustain a notably higher rate of energy intake between 2014 and 2024 as compared to a scenario in which sea urchins and mussel populations remained stable (Fig. 5D).

### Sea otter population dynamics

**Fig. 6.**
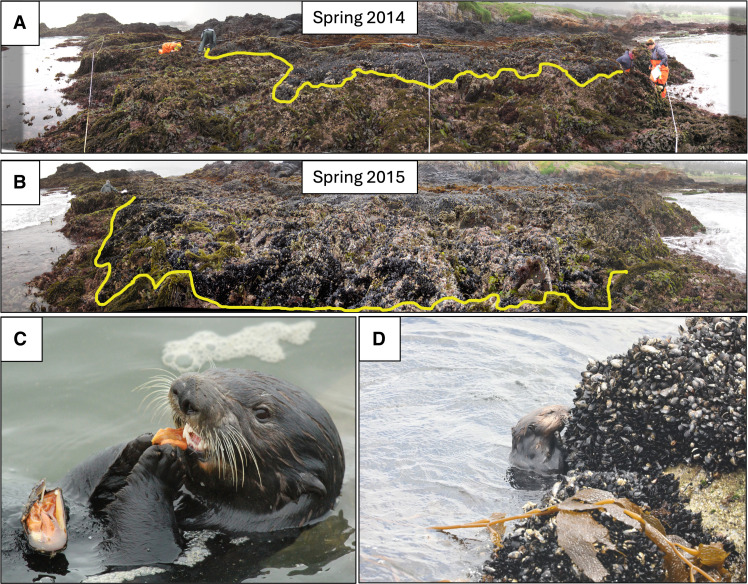
Mussel expansion and sea otter foraging. In (**A**) distribution of mussels (*M. californianus*) before the sea star wasting event at Stillwater cove, California, USA. In (**B**), mussel cover expands lower in the rocky intertidal following the collapse of *P. ochraceus*. Images in (A) and (B) were obtained from approximately the same location pre- and post-sea star wasting. The yellow line depicts the approximate lower distribution of mussels in each photo. (**C**) Sea otter foraging on a mussel. (**D**) Sea otter foraging on a patch of mussels attached to a rocky outcrop extending into the lower intertidal. Photo credits: [(A) and (B)], Multi-Agency Rocky Intertidal Network Research Group at University of California, Santa Cruz; [(C) and (D)], © Monterey Bay Aquarium.

The local sea otter population showed a notable increase in density beginning in 2014, which coincided with sea urchin outbreaks in the subtidal, but was likely sustained by later (2016) increases in mussel abundance within the rocky intertidal (Figs. 2A and 6). Along the Monterey Peninsula, total sea otter abundance grew from a decade (2000–2012) average of 373 ± 49 to 535 ± 91 independents (i.e., individuals no longer dependent on their mother for survival) during 2014–2024, indicating a strong numerical response to increases in the availability of mussels and sea urchins.

## DISCUSSION

This study advances our understanding of keystone predation by revealing keystone interdependence as a mechanism of cross-ecosystem coupling and community regulation. Our results demonstrate that prey release from a keystone predator in one system can provide a surplus that supports another keystone predator in an adjacent system and that these changes can be correlated with extreme events including epizootics and climate-driven marine heatwaves (MHWs). Traditional models of keystone predation and trophic cascades often focus on within-ecosystem dynamics, but our findings suggest that predator-prey interactions can have cascading effects that transcend ecosystem boundaries, reinforcing the need to consider spatially and functionally connected food webs. The concept of keystone interdependence builds on and expands classical ecological theory by demonstrating that the indirect effects of a keystone predator’s decline can extend across adjacent ecosystems, altering the distribution of energy transfer and associated mechanisms of community regulation.

In the two decades preceding the sea star wasting event that decimated populations of the keystone predator, *P. ochraceus,* the rocky intertidal and adjacent subtidal kelp forests were both under relatively strong top-down control (*19*, *27*). During this period, prey limitation in the subtidal contributed in part to sea otters reaching a local population equilibrium (*19*). However, our results revealed that the loss of *Pisaster* following a coastwide sea star wasting event in 2013 resulted in increased cover, size structure, and expansion of mussels (*M. californianus*) within the intertidal zone. Consequently, sea otters increased their dietary intake (e.g., per capita and total) of mussels in the rocky intertidal and sea urchins in the subtidal (*7*), which contributed to local population growth. We therefore infer that keystone interdependence can occur through a series of indirect trophic interactions, and these processes can drive community restructuring across ecosystems.

Understanding the ecological histories of systems is important for interpreting the contexts that determine the relative influence of top-down versus bottom-up community regulation (*7*, *26*, *28*, *29*). While *Pisaster* is often considered a keystone species (*16*, *30*), physical disturbance and recruitment limitation can also prevent space dominance of primary consumers within rocky intertidal communities (*31*). Similarly, the community-wide effects of apex predation by sea otters manifest differently across their northern Pacific range in response to local and regional environmental conditions (*7*). For example, in the Aleutian Islands, where sea otters repatriated areas dominated by herbivorous sea urchins that were presumably low in energetic profitability, predation reduced sufficient biomass of sea urchins to indirectly enhance the recovery of kelp forests (*18*). However, in central California, where an outbreak of sea urchins occurred within this predator’s established range, sea otters indirectly enhanced the resistance of remnant forests by targeting gravid sea urchins despite patchy sea urchin barrens (*7*).

In our study, we assume that pre-2013 community configurations of the rocky intertidal and subtidal kelp forests were both under strong top-down control and that two pathways likely initiated community structuring. First, the 2013 sea star wasting event that decimated *Pisaster* in the rocky intertidal led to an increase in mussel abundance, although the increase in mussels lagged for approximately 3 years due to demographics such as recruitment and growth. Second, in this system, a large Pacific MHW occurred from 2014 to 2016 (*32*). The MHW resulted in declines in kelp productivity (and drift kelp production) that led to local outbreaks of purple sea urchins (*Strongylocentrotus purpuratus*) in the subtidal beginning in 2014 (*25*), and these herbivore outbreaks were later reinforced by increases in sea urchin recruitment (*33*). Our results suggest that increases in prey abundance resulting from demographic (mussels) and behavioral (sea urchins) shifts can lead to community restructuring.

While our results indicate that increased sea otter abundance after 2014 is explained in part by energetic gain from mussels, sea urchin consumption also substantially increased during the same period. In the 2 to 3 years following the large Pacific MHW, the dietary contribution of sea urchins rapidly increased. However, a sharp increase in mussel consumption occurred in 2016 and continued to increase over time, which ultimately became a major source of caloric intake in the post-sea star wasting period. The delayed contribution of mussels to sea otter diets is probably due to population demographics, such that mussel expansion into the lower rocky intertidal was limited by recruitment, growth, and survivorship (*34*, *35*) as well as spatial expansion to an area low enough where they were more accessible by sea otters and reaching a size that would be attractive to a large proportion of the sea otter population. By contrast, the sea urchin outbreak was initiated by a behavioral shift that coincided with the MHW and occurred over a much shorter temporal scale (*25*, *26*). This initial outbreak of sea urchins prompted a more immediate prey shift by foraging sea otters. However, sustained year-over-year increases in mussel consumption are reflective of their population growth within the rocky intertidal, which ultimately became a major energetic source for sea otters.

One alternative hypothesis for observed increases in both mussel consumption and sea otter abundance is movement of sea otters into the study area. While Smith *et al.* (*7*) found that movement from areas to the north and south was not strongly correlated with increases in abundance during the same time period, it is possible that some individuals may have relocated into the study area from areas offshore that were beyond observable viewing range. However, this would further support that increases in mussels and sea urchins sustained elevated levels of energetic intake, even for a larger population of sea otters. Nonetheless, despite increases in the dietary intake of mussels and sea urchins, the overall energy intake at the level of the local sea otter population declined in the post-sea star wasting period, apparently due to declines in other kelp forest-associated herbivores such as abalone, crabs, stars and cephalopods. The results of our behavioral-bioenergetic model suggest that the increased availability of sea urchins and then mussels during this period allowed for a higher net rate of energy intake than would have been possible otherwise, particularly for younger sea otters. Other factors supporting the persistence of elevated sea otter numbers include individual foraging strategies that diversify effects on local prey populations (*19*), redistribution from less observable areas, or increased reliance on nearby alternative habitats such as soft-sediment areas and estuaries.

Keystone interdependence may contribute to community restructuring through a series of predator behavioral responses. The results of our alternative energetic intake model indicated that the increase in mussels is not expected to affect sea urchin consumption because sea otters continue to prefer high quality sea urchin patches over mussels when encountered. This suggests that the increase in mussel abundance did not necessarily decrease the effect of top-down control of sea urchins. We also recognize that there are potential energetic trade-offs between foraging on mussels in the rocky intertidal versus sea urchins in the subtidal, although further studies could elucidate the broader cross-ecosystem consequences of these energetic budgets (*36*). However, given the elevated abundance of sea otters in this region, an eventual decline in mussel cover may result in predation on less profitable sea urchins or a switch to other invertebrate prey. Nonetheless, overall sea otter foraging on both sea urchins and mussels increased (*7*), resulting in positive indirect benefits to kelp forest persistence.

Although sea otters benefited from increased mussel abundance, this prey surplus may only be temporary, which could affect the persistence of the current ecosystem state. Because mussels appear to have reached a size-class refuge from predation by sea stars (*37*), a return of the rocky intertidal community to the prior (pre-2013) state may be slow. This larger size class of mussels, however, will likely be targeted by sea otters that may also contribute to eventual shifts in the rocky intertidal state. We hypothesize that once *Pisaster* recovers and reestablishes control over the lower distribution of mussels, this prey surplus for sea otters may no longer persist, resulting in a return of the system to its pre-2013 configuration. In addition, although sea otters do forage on sea stars when available, the contribution of sea stars to sea otter diets is disproportionately low and would not have resulted in the control of sea star populations through direct predation (*22*).

Future studies on keystone species should explicitly consider interecosystem linkages and their potential to mediate resilience or transformations in response to perturbations. Further studies are needed to determine how broadly applicable keystone interdependence is in other systems, particularly in regions where energy transfer occurs across ecosystem boundaries through direct and indirect species interactions [e.g., (*1*, *3*–*5*)]. As climate-driven shifts continue to reshape marine communities, the dynamics of keystone interdependence may play an increasingly important role in linking ecosystems in novel and complex ways. Understanding these processes will be critical for refining ecological theory, improving predictive models of ecosystem change, and informing conservation and management strategies that account for cross-ecosystem feedbacks.

## MATERIALS AND METHODS

To test our four hypotheses, we synthesized data spanning two decades of long-term rocky intertidal monitoring, sea otter population surveys, and spatially explicit observations of sea otter foraging behavior.

### Study area

This study was conducted along the Monterey Peninsula, California, USA. The study area consists of several kilometers of expansive rock that extends from the intertidal zone into subtidal kelp forests. Throughout the region, sea otters consume a variety of invertebrate prey (fig. S2). In the rocky intertidal, habitat-forming mussels are consumed by their main predator, *Pisaster* (Fig. 1C)*.* The study area has multiple long-term monitoring programs, which allows for comparisons pre- and post-2013 sea star wasting event and spatial resolution of intertidal and subtidal habitats used by sea otters, *Pisaster*, and mussels.

### Sea star and mussel dynamics in the rocky intertidal

To test the hypothesis that the sudden collapse of *Pisaster* in the rocky intertidal resulted in mussel expansion (i.e., increased cover and distribution into the lower tidal zone, Fig. 1B), we analyzed temporal variation in abundance using rocky intertidal monitoring data (*38*). The Multi-Agency Rocky Intertidal Network is a consortium of federal, state, university, and private organizations that collect biological community-level data at sites distributed from Southeast Alaska to Mexico (for detailed sampling methods, see Supplementary Methods). For our analyses, we selected four sites located within the study area (Fig. 2G). These four sites had over two decades of monitoring data spanning the periods before, during, and after the sea star wasting event.

To determine temporal dynamics, we explored annual trends in the mean cover of mussels and abundance of *Pisaster* across the time series of our four study sites. First, we examined mean annual trends in mussel cover across all sites using a cubic spline fit across all sites and years. For *Pisaster,* we modeled three time periods as separate functions because some years were not sampled. We first fitted the 2001–2007 period using a Gompertz growth function (*39*), then extrapolated the trend to the 2008–2012 period. Given the abrupt onset of the 2013 sea star wasting event, and the near collapse of this population, we fit years 2013 and beyond using a cubic spline. If the loss of *Pisaster* is associated with an increase in mussel cover, then the post-2013 slope of mussels should be inversely related to the slope of *Pisaster*.

We comparatively evaluated the spatial distribution of mussels at each long-term monitoring site before (2009–2012) versus after (2013–2021) the sea star wasting event. Rocky intertidal surveys consist of a grid-sampling design at each site, with a baseline transect 20 to 30 m long extending parallel to the water in the high intertidal and 11 perpendicular transects that extend from the baseline toward the lower tide zone. This sampling framework allowed for comparison of presence/absence of mussels across space and through time. For each site, we plotted the presence of mussels observed at every point along the grid for all years before and after the sea star wasting event. We then plotted the frequency distribution of mussel from the base transect to the MLLW level (m; i.e., distance from baseline transect), size (mm), and total percent cover across the two time periods and evaluated significance for each of these three variables using a *t* test.

### Sea otter foraging observations

Sea otter diets and foraging behavior were evaluated using long-term foraging surveys conducted within the study area from 2007 to 2023. Surveys consisted of spatially explicit observations of longitudinal sequences of 20 or more feeding dives made by randomly selected individual sea otters (a feeding bout). For each feeding bout, observers recorded dive and surface intervals, dive outcomes (whether prey was successfully captured), prey type (to the lowest taxonomic level possible), size and number of each prey item captured per dive, and other associated data [(*19*, *40*) and the Supplementary Methods].

To identify trends in sea otter dietary composition and energetic intake over time, we combined foraging survey data with published information on taxa-specific caloric densities and size-biomass relationships for sea otter prey (*19*). To simplify analyses, we first categorized prey species into 1 of 11 district prey types (table S2). We then used a previously described Bayesian inference framework [Sea Otter Foraging Analysis (SOFA)] to analyze the data to account for sampling uncertainty and potential biases in the observational data caused by nonrandom probabilities of prey identification related to prey size and taxa (*19*). Fitting the SOFA model to the observed dataset resulted in bias-corrected posterior estimates of foraging effort allocation (𝜂*_i,t_*, the proportion of all feeding dives allocated to prey type *i* in year *t*), the rate of energetic intake (*E*, kcal/min) for dives allocated to each prey type, and realized diet composition (the relative contribution of each prey type to consumed biomass).

### Sea otter caloric intake from sea urchins and mussels

We used the results of the SOFA model as data inputs for a secondary model designed to evaluate the implications of observed increases in mussels and urchins for sea otter bioenergetics. We began with the core assumption that sea otters will tend to allocate foraging effort among potential prey types to maximize their realized rate of energy intake over time, an assumption well supported by many previous studies [e.g., (*19*, *41*–*46*)]. Our model describes dynamics of latent parameters δ*_i,t_*, the effective density of each prey type *i* in year *t*. We define effective density as the instantaneous encounter rate with suitable patches of prey type *i* for a foraging sea otter (we assume suitability is determined by prey-specific selection biases based on size, quality, or other prey attributes). Another key parameter is $E_{i}$, the average expected rate of energy return for an otter feeding within a suitable patch of prey type *i*. For computational tractability we estimate the log of both parameters [μ=log(E) and Δ=log(δ)], and we use an auto-regressive formulation for estimating $\Delta_{i,t}$ to account for temporal autocorrelation in prey population abundance

$$\Delta_{i,t}∼\text{normal}\left(\Delta_{i,t-1},\sigma_{D,i}\right)$$ (1)

where $\sigma_{D,i}$ is a variance parameter to be estimated by the model. Because parameter δ represents an instantaneous rate, we calculate the finite probability of encountering a patch of prey type i during a feeding dive $\left(\lambda_{i,t}\right)$ as

$$\lambda_{i,t}=1-\text{exp}\left(-\delta_{i,t}\right)$$ (2)

We next define a derived parameter $\left(\pi_{i,t}\right)$ representing the joint probability of encountering a patch of prey type i and not encountering a patch of a more profitable prey type during a feeding dive

$$\text{log}\left(\pi_{i,t}\right)=\text{log}\left(\lambda_{i,t}\right)+\sum_{j}\text{log}\left(1-\lambda_{j,t}\cdot W_{i,j}\right)$$ (3)

where j is the array of prey types excluding i and $W_{i,j}$ is a switch variable set to 1 if $E_{j}>E_{i}$, otherwise set to 0. Assuming that sea otters make optimal decisions as to which patches to forage in, the average proportional allocation of foraging effort among *K* potential prey types in year $t\left(\eta_{t}=<\eta_{1,t},\eta_{2,t},\ldots \eta_{K,t}>\right)$ will be proportional to vector $<\pi_{1,t},\pi_{2,t},\ldots \pi_{K,t}>\text{where}\;{\Sigma \pi }_{i,t}=1$.

To fit the model, we used Markov Chain Monte Carlo (MCMC) methods to relate predictions of the process model to “observed” (bias-corrected) data variables generated by the SOFA analysis. Specifically, we assume that observed log mean rates of energy intake for each prey type in each year are normally distributed around the true mean values for each prey type

$$\mu_{\text{obs},i,t}∼\text{normal}\left(\mu_{i},\sigma_{E,i}\right)$$ (4)

where $\sigma_{E,i}$ is a prey-specific measurement variance parameter to be estimated by the model. We further assume that observed annual estimates of foraging effort allocation are described by a Dirichlet distribution with parameters determined by $\pi_{i,t}$

$$\eta_{\text{obs},t}∼\text{Dirichlet}\left(\tau_{t}\cdot <\pi_{1,t},\pi_{2,t}\ldots \pi_{K,t}>\right)$$ (5)

where $\tau_{t}$ is a Dirichlet precision parameter calculated before model fitting by iteratively drawing arrays of $\eta_{i,t}$ from the joint posterior distributions generated by the SOFA model and using maximum likelihood methods to fit a Dirichlet distribution to the resulting matrix (using R library “dirichlet”).

We used Stan software to code and fit the above-described model within the R programming environment (*47*, *48*). We used uninformative Cauchy or half-Cauchy priors for all model-estimated parameters and conducted standard model diagnostic procedures to ensure model convergence and goodness of fit (Supplementary Methods). We then used the fitted model to estimate several derived parameters of interest. First, we calculated mean overall rate of energy intake for an optimally foraging sea otter in year t

$${\bar{E}}_{t}=\sum_{i}\pi_{i,t}\cdot E_{i}$$ (6)

We next evaluated three alternative scenarios of prey dynamics. The first alternative scenario assumed that sea urchin densities did not increase after 2012 but instead remained within the range of densities observed before 2013: To achieve this, we manually adjusted the values of $\delta_{i,t}$ for urchins in years after 2012, drawing the new values from a normal distribution with mean and variance calculated from the estimated values for 2007–2012 (all other prey densities were assumed to vary as observed). The second alternative scenario assumed that mussel densities did not increase but instead remained within the range of densities observed before 2013: To achieve this, we manually adjusted the values of $\delta_{i,t}$ for mussels in years after 2012, drawing the new values from a normal distribution with mean and variance calculated from the estimated values for 2007–2012. The third scenario assumed that both urchins and mussels did not increase but instead remained within the range of densities observed before 2013. For each alternative scenario, we iteratively resolved the process model described by Eqs. 1 to 5, drawing all other parameters from the joint posterior distributions generated by the fitted model, and then recalculated the mean expected overall rate of energy intake under the scenario by resolving Eq. 6. This approach allowed patch encounter probabilities (Eqs. 2 and 3) to be recalculated appropriately for each scenario of prey abundance, thus ensuring that projected foraging effort allocations and energy intake rates reflected optimal foraging decisions under each scenario. We also calculated expected per-capita predation rates ($P_{i,t}$ = mean number of items of prey type i consumed per minute by a foraging otter in year *t*) for mussels and urchins

Pi,t=πi,t⋅EiGi (7)

where $G_{i}$ is the mean Kcal value for an item of prey type *i*, calculated as the product of mean caloric density and mean edible biomass (calculated from mean prey size using published size-biomass functional relationships). Note that variance in $G_{i}$ was propagated by iteratively drawing prey sizes and caloric densities from their posterior distributions generated by the SOFA analysis.

We calculated mean values and 95% credible intervals for all statistics based on posterior projections of the alternative scenarios. Comparing results among alternative scenarios allowed us to explore how changes in availability of mussels and urchins affected overall energy intake rates and prey-specific predation rates by foraging sea otters.

### Sea otter population dynamics

To examine annual trends in southern sea otter abundance throughout the study area, we used census data from 2000 to 2023 (*49*). Sea otter census data were collected annually from 2000 to 2023, with the exception of years 2011 and 2020. We calculated the total abundance of independent sea otters (those not dependent on their mothers for survival) throughout the study area and examined trends over time.

## Supplementary Materials

### This PDF file includes:

Supplementary Methods Figs. S1 and S2 Tables S1 and S2 References
