# Supplementary material for: Keystone interdependence: Sea otter responses to a prey surplus following the collapse of a rocky intertidal predator
Source: Sci Adv. 2025 Apr 30;11(18):eadu1028. doi: 10.1126/sciadv.adu1028 (PMC12042895; doi:10.1126/sciadv.adu1028)
Supplement: Supplementary file 1 — Supplementary Methods Figs. S1 and S2 Tables S1 and S2 References [file sciadv.adu1028_sm.pdf]

Science Advances
AAAS

Supplementary Materials for

Keystone interdependence: Sea otter responses to a prey surplus following the collapse of a rocky intertidal predator

Joshua G. Smith *et al.*

Corresponding author: Joshua G. Smith, josgraysmith@gmail.com

Sci. Adv. **11**, eadu1028 (2025)
DOI: 10.1126/sciadv.adu1028

This PDF file includes:

Supplementary Methods
Figs. S1 and S2
Tables S1 and S2
References

Supplementary methods

Rocky intertidal sampling design

Long-term monitoring and biodiversity surveys in rocky intertidal habitats are conducted annually as part of the Multi-Agency Rocky Intertidal Network (MARINe; *38*, *50*). These surveys are conducted at more than 100 long-term monitoring sites along the West Coast of the United States and target 25 key species and species assemblages. Long-term monitoring surveys use fixed sampling areas to document changes in percent cover, or abundance of targeted species or species assemblages. This fixed-plot approach allows the dynamics of rocky intertidal species to be monitored with reasonable sampling effort and provides sufficient statistical power to detect changes over space or time. The MARINe survey methods can be divided into three sampling approaches. First, permanent photoplots (called photoplots because, in addition to sampling these plots in the field, photos are taken of each plot when sampling) are used to monitor percent cover of organisms within target species assemblages. Plots are established for a given species assemblage if overall cover at a site is sufficient for monitoring. In general, 5 replicate plots (50 x 75 cm) are placed in a stratified random manner throughout the target species' occupied tide zone. Plots are photographed in the field and are scored for percent cover using a grid of 100 points. A species, higher taxon, or substrate located below each of the 100 points is identified and recorded. Layering is generally scored separately, so the total cover is 100 percent. When assessing layering the spatial position is also noted such that the top and bottom layers are recorded for each location on the grid. This provides extra data that can be used to gather information on species associations and small-scale spatial changes over time. Second, transects are used to monitor percent cover of surfgrass, kelps, and other algae. In general, three replicate transects are established for each target species. Transects are generally 10 m long and percent cover is estimated by recording the taxa or substrate occurring under each 10 cm interval (for a total of 100 points). Third, the number and size of a specific organism found in larger permanent plots at a given site are counted. The core species (i.e., specific subset selected for their ecological, economic, or cultural importance) are assessed using this approach. See MARINe et al. (*38*) for data and further information on sampling design.

Sea otter census survey

Sea otter habitat is considered to extend offshore from the mean low tide line and out to the 60 m isobath. This depth range includes over 99% of sea otter feeding dives, based on dive-depth data from radio tagged sea otters (*19*, *51*, *52*). The southern sea otter distribution in California occurs in this band of potential habitat stretching along the coast. Since 1983, except 2011 and 2020, southern sea otter population surveys have been conducted annually in the spring throughout the range (approximately 600 km) using a combination of shore-based and aerial survey techniques. Shore-based observers use binoculars and spotting scopes to count sea otters from accessible stretches of coastline. The remaining areas are usually counted from a fixed-wing aircraft. The nearshore stretch of the Monterey Peninsula is among the areas surveyed by shore.

The survey records the total otter numbers, the number of dependent pups, and the number of independents (adults and subadults), observed. All counts are entered into a geographic information system and aggregated into spatial bins divided in approximately 500-

meter widths along a smoothed 5 fathom bathymetric contour line along the California shore, defined as ATOS (As-The-Otter-Swims) bins (*49*). To reduce the influence of anomalously high or low counts during any particular year, the raw data are smoothed by averaging the counts spatially (in 10-km moving windows) and temporally (as 3-year running averages) to assess whether the local or whole population has grown or declined. The Monterey Peninsula data correspond to smoothed averages centered around ATOS 362 to 449.

Sea otter foraging surveys

Sea otter foraging surveys were conducted opportunistically on tagged and untagged wild sea otters using previously established methods (*19*, *46*, *53*). Observations were collected during daylight hours from shore using Questar 50-80X telescopes (Questar Corporation, New Hope, PA). Once a feeding sea otter was identified, observers would initiate collecting a foraging bout which consisted of the focal otter making continuous dives underwater and returning to the surface to either consume the collected prey or dive again if unsuccessful. Data collection continued until the animal went out of view or stopped foraging (number of dives per bout: 5-55).

For each dive, the sea otter's location was estimated based on GPS coordinates, compass bearing, distance from observers, and distance from shore. Other variables collected included the type of habitat in which the sea otter was foraging (e.g., in kelp, off emergent rocks, in open water, etc.), dive duration (duration of time at which an otter is underwater), surface intervals (duration of time that an otter is at the surface), and dive outcome (indicator of whether or not prey was successfully captured). For dives that had a successful capture of prey recorded, prey were identified to the lowest taxonomic level possible. Each prey item was counted and sized based on 1.67 cm size-class categories identified in relation to an average sea otter fore-paw width of 5 cm.

Supplementary figures

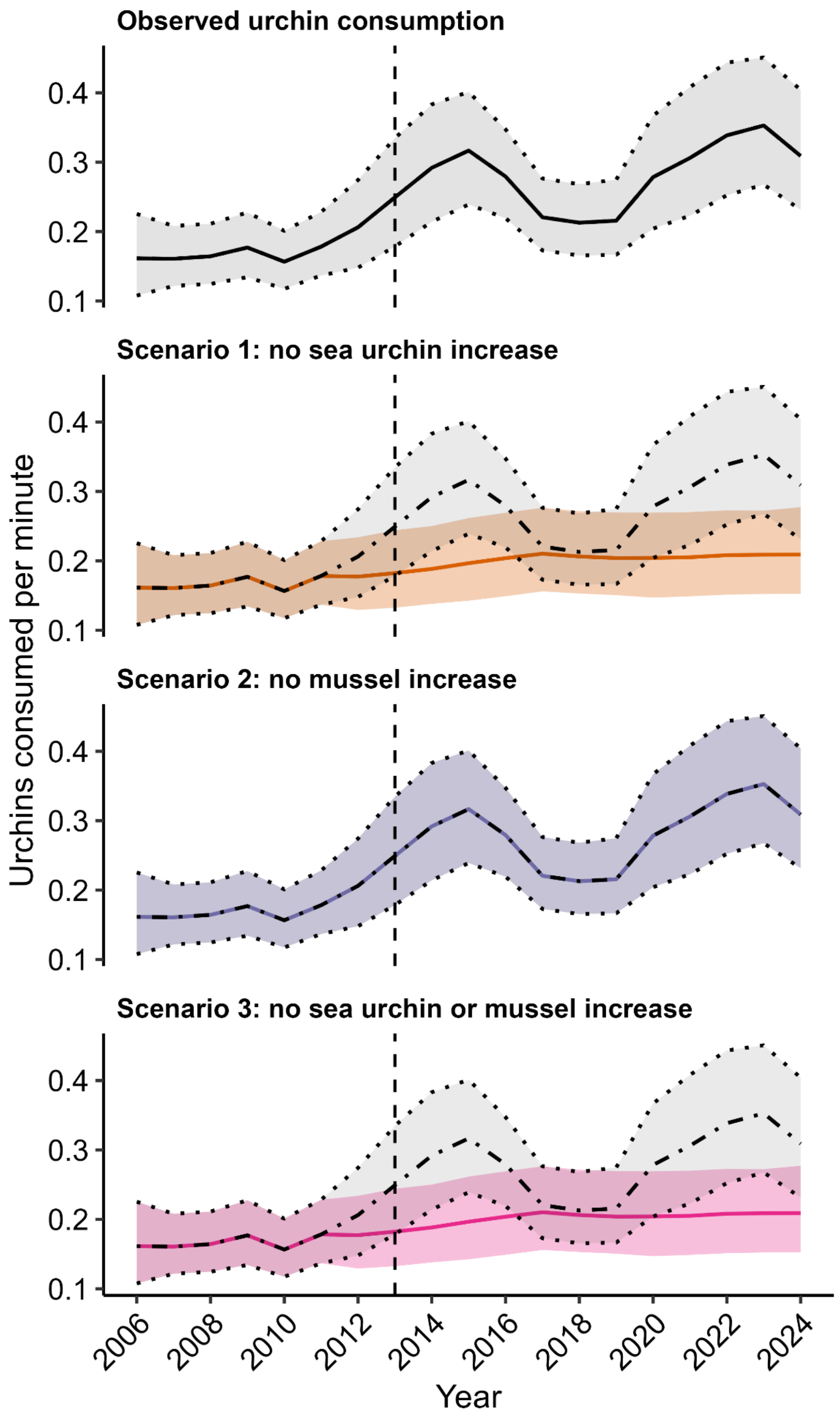

Fig. S1. Trends in sea urchins consumed per minute, as estimated from data collected in field surveys and analyzed using a Bayesian hierarchical model. Top panel shows trends reflecting observed sea urchin consumption. Lower panels compare observed trends (light grey shaded

band) to predicted trends under 3 alternative scenarios: 1) no post-2012 increase in urchin density; 2) no post-2012 increase in mussel density; and 3) no post-2012 increase in urchins or mussels. For each plot the shaded bands indicate 95% CI and solid lines indicate point estimates of energy intake rates.

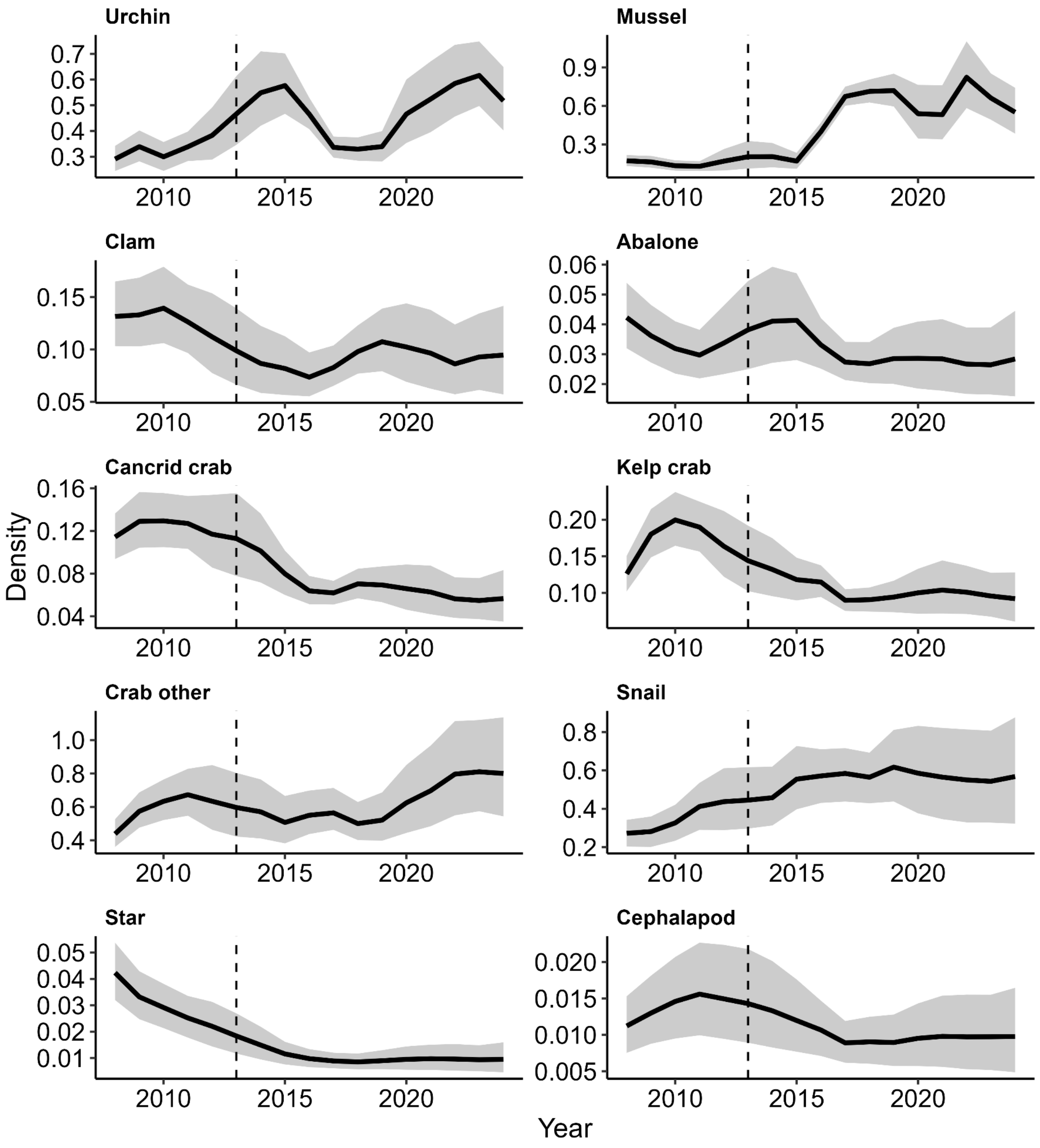

Fig. S2. Estimated trends in the density of alternative prey over time. Each panel represents a different prey taxa, with relative density (instantaneous encounter rates for prey patches of each type by foraging otters) shown as solid lines and 95% confidence intervals as shaded ribbons. Some confidence intervals were truncated at a maximum value of 2 to ease visualization of the underlying trends. The dashed vertical line in 2013 marks the onset of the sea star wasting (SSW) event. Data are derived from a Bayesian hierarchical model estimating variation in prey encounter rates and the associated optimal allocation of sea otter foraging effort over time.

Supplementary tables

Table S1. Metadata for products used in analyses

Product	Description	Source
MARINe	Rocky intertidal long-term monitoring and biodiversity surveys	https://marine.ucsc.edu/
Sea otter census	Annual California Sea Otter Census	https://www.sciencebase.gov/catalog/item/5601b6dae4b03bc34f5445ec
Sea otter foraging	Sea otter foraging surveys	Monterey Bay Aquarium Sea Otter Research and Conservation

Table S2. Sea otter foraging prey table. Prey table includes 11 categories of sea otter prey, mean energy intake rates (E) with credible intervals (+ or - 95%), and mean encounter rates (effort) pre and post-2013 with standard deviations (S.D.).

Prey type	E (Kcal/min)	E s.d.	Pre-2013 effort	Pre-2013 effort s.d.	Post-2013 effort	Post-2013 effort s.d.
Sea urchin	7.34	0.32	17.29	1.71	28.23	4.77
Mussel	6.09	0.57	6.95	1.51	17.87	7.56
Clam	7.44	0.52	7.72	0.56	6.11	0.78
Abalone	21.28	0.47	3.72	0.74	3.04	0.52
Cancrid crab	11.84	0.23	10.59	0.59	6.50	1.51
Kelp crab	8.12	0.49	12.09	2.14	8.86	1.13
Crab other	5.27	0.43	15.88	1.29	11.64	1.53
Snail	5.02	0.50	6.52	0.76	7.00	1.53
Sea star	15.81	0.88	3.43	1.15	1.03	0.27
Cephalopod	27.51	0.21	1.32	0.18	1.05	0.18
Other	3.75	0.17	14.49	2.37	8.67	2.35

REFERENCES AND NOTES

1. G. A. Polis, W. B. Anderson, R. D. Holt, Toward an integration of landscape and food web ecology: The dynamics of spatially subsidized food webs. *Annu. Rev. Ecol. Syst.* **28**, 289–316 (1997).

2. S. A. Navarrete, E. A. Wieters, B. R. Broitman, J. C. Castilla, Scales of benthic–pelagic coupling and the intensity of species interactions: From recruitment limitation to top-down control. *Proc. Natl. Acad. Sci. U.S.A.* **102**, 18046–18051 (2005).

3. G. H. Roffler, C. E. Eriksson, J. M. Allen, T. Levi, Recovery of a marine keystone predator transforms terrestrial predator–prey dynamics. *Proc. Natl. Acad. Sci. U.S.A.* **120**, e2209037120 (2023).

4. J. A. Walter, K. A. Emery, J. E. Dugan, D. M. Hubbard, T. W. Bell, L. W. Sheppard, V. A. Karatayev, K. C. Cavanaugh, D. C. Reuman, M. C. N. Castorani, Spatial synchrony cascades across ecosystem boundaries and up food webs via resource subsidies. *Proc. Natl. Acad. Sci. U.S.A.* **121**, e2310052120 (2024).

5. D. E. Schindler, M. D. Scheuerell, Habitat coupling in lake ecosystems. *Oikos* **98**, 177–189 (2002).

6. J. W. Terborgh, Toward a trophic theory of species diversity. *Proc. Natl. Acad. Sci. U.S.A.* **112**, 11415–11422 (2015).

7. J. G. Smith, J. Tomoleoni, M. Staedler, S. Lyon, J. Fujii, M. T. Tinker, Behavioral responses across a mosaic of ecosystem states restructure a sea otter–urchin trophic cascade. *Proc. Natl. Acad. Sci. U.S.A.* **118**, e2012493118 (2021).

8. T. L. Rogers, S. B. Munch, S. D. Stewart, E. P. Palkovacs, A. Giron-Nava, S. S. Matsuzaki, C. C. Symons, Trophic control changes with season and nutrient loading in lakes. *Ecol. Lett.* **23**, 1287–1297 (2020).

9. T. H. Larsen, N. M. Williams, C. Kremen, Extinction order and altered community structure rapidly disrupt ecosystem functioning. *Ecol. Lett.* **8**, 538–547 (2005).

10. J. E. Hewitt, S. F. Thrush, P. D. Dayton, Habitat variation, species diversity and ecological functioning in a marine system. *J. Exp. Mar. Biol. Ecol.* **366**, 116–122 (2008).

11. P. Kratina, H. S. Greig, P. L. Thompson, T. S. A. Carvalho-Pereira, J. B. Shurin, Warming modifies trophic cascades and eutrophication in experimental freshwater communities. *Ecology* **93**, 1421–1430 (2012).

12. L. Pecuchet, M. Lindegren, S. Kortsch, J. Całkiewicz, I. Jurgensone, P. Margonski, S. A. Otto, I. Putnis, S. Strāķe, M. C. Nordström, Spatio-temporal dynamics of multi-trophic communities reveal ecosystem-wide functional reorganization. *Ecography* **43**, 197–208 (2020).

13. C. Bello, M. Schleuning, C. H. Graham, Analyzing trophic ecosystem functions with the interaction functional space. *Trends Ecol. Evol.* **38**, 424–434 (2023).

14. M. H. Carr, D. C. Reed, Chapter 17: Shallow rocky reefs and kelp forests*, in Ecosystem of California, H. Mooney and E. Zavaleta*, Eds. (Univ. of California Press, 2016), pp. 311–336.

15. A. I. Borthagaray, A. Carranza, Mussels as ecosystem engineers: Their contribution to species richness in a rocky littoral community. *Acta Oecol.* **31**, 243–250 (2007).

16. R. T. Paine, Intertidal community structure. *Oecologia* **15**, 93–120 (1974).

17. S. B. Traiger, J. L. Bodkin, H. A. Coletti, B. Ballachey, T. Dean, D. Esler, K. Iken, B. Konar, M. R. Lindeberg, D. Monson, B. Robinson, R. M. Suryan, B. P. Weitzman, Evidence of increased mussel abundance related to the Pacific marine heatwave and sea star wasting. *Mar. Ecol.* **43**, e12715 (2022).

18. J. A. Estes, J. F. Palmisano, Sea otters: Their role in structuring nearshore communities. *Science* **185**, 1058–1060 (1974).

19. M. T. Tinker, G. Bentall, J. A. Estes, Food limitation leads to behavioral diversification and dietary specialization in sea otters. *Proc. Natl. Acad. Sci. U.S.A.* **105**, 560–565 (2008).

20. S. A. Navarrete, B. A. Menge, Keystone predation and interaction strength: Interactive effects of predators on their main prey. *Ecol. Monogr.* **66**, 409–429 (1996).

21. J. P. Suraci, M. Clinchy, L. Y. Zanette, C. M. A. Currie, L. M. Dill, Mammalian mesopredators on islands directly impact both terrestrial and marine communities. *Oecologia* **176**, 1087–1100 (2014).

22. M. T. Tinker, J. A. Tomoleoni, B. P. Weitzman, M. Staedler, D. Jessup, M. J. Murray, M. Miller, T. Burgess, L. Bowen, A. K. Miles, N. Thometz, L. Tarjan, E. Golson, F. Batac, E. Dodd, E. Berberich, J. Kunz, G. Bentall, J. Fujii, T. Nicholson, S. Newsome, A. Melli, N. LaRoche, H. MacCormick, A. Johnson, L. Henkel, C. Kreuder-Johnson, P. Conrad, "Southern sea otter (Enhydra lutris nereis) population biology at Big Sur and Monterey, California—Investigating the consequences of resource abundance and anthropogenic stressors for sea otter recovery" (2019–1022, U.S. Geological Survey, 2019); https://doi.org/10.3133/ofr20191022.

23. M. T. Tinker, J. L. Yee, K. L. Laidre, B. B. Hatfield, M. D. Harris, J. A. Tomoleoni, T. W. Bell, E. Saarman, L. P. Carswell, A. K. Miles, Habitat features predict carrying capacity of a recovering marine carnivore. *J. Wildl. Manag.* **85**, 303–323 (2021).

24. C. D. Harvell, D. Montecino-Latorre, J. M. Caldwell, J. M. Burt, K. Bosley, A. Keller, S. F. Heron, A. K. Salomon, L. Lee, O. Pontier, C. Pattengill-Semmens, J. K. Gaydos, Disease epidemic and a marine heat wave are associated with the continental-scale collapse of a pivotal predator (*Pycnopodia helianthoides*). *Sci. Adv.* **5**, eaau7042 (2019).

25. J. G. Smith, M. T. Tinker, Alternations in the foraging behaviour of a primary consumer drive patch transition dynamics in a temperate rocky reef ecosystem. *Ecol. Lett.* **25**, 1827–1838 (2022).

26. J. G. Smith, D. Malone, M. H. Carr, Consequences of kelp forest ecosystem shifts and predictors of persistence through multiple stressors. *Proc. Biol. Sci.* **291**, 20232749 (2024).

27. M. M. Moritsch, Expansion of intertidal mussel beds following disease-driven reduction of a keystone predator. *Mar. Environ. Res.* **169**, 105363 (2021).

28. B. A. Menge, J. P. Sutherland, Community regulation: Variation in disturbance, competition, and predation in relation to environmental stress and recruitment. *Am. Nat.* **130**, 730–757 (1987).

29. E. S. Poloczanska, C. J. Brown, W. J. Sydeman, W. Kiessling, D. S. Schoeman, P. J. Moore, K. Brander, J. F. Bruno, L. B. Buckley, M. T. Burrows, C. M. Duarte, B. S. Halpern, J. Holding, C. V. Kappel, M. I. O'Connor, J. M. Pandolfi, C. Parmesan, F. Schwing, S. A. Thompson, A. J. Richardson, Global imprint of climate change on marine life. *Nat. Clim. Change* **3**, 919–925 (2013).

30. B. A. Menge, E. L. Berlow, C. A. Blanchette, S. A. Navarrete, S. B. Yamada, The keystone species concept: Variation in interaction strength in a rocky intertidal habitat. *Ecol.l Monogr.* **64**, 249–286 (1994).

31. P. K. Dayton, Toward an understanding of community resilience and the potential effects of enrichments to the benthos at McMurdo Sound, Antarctica, in *Proceedings of the colloquium on conservation problems in Antarctica* (1972), vol. 96.

32. E. Di Lorenzo, N. Mantua, Multi-year persistence of the 2014/15 North Pacific marine heatwave. *Nat. Clim. Change* **6**, 1042–1047 (2016).

33. D. K. Okamoto, S. C. Schroeter, D. C. Reed, Effects of ocean climate on spatiotemporal variation in sea urchin settlement and recruitment. *Limnol. Oceanogr.* **65**, 2076–2091 (2020).

34. B. A. Menge, Relative importance of recruitment and other causes of variation in rocky intertidal community structure. *J. Exp. Mar. Biol. Ecol.* **146**, 69–100 (1991).

35. D. R. Schiel, The structure and replenishment of rocky shore intertidal communities and biogeographic comparisons. *J. Exp. Mar. Biol. Ecol.* **300**, 309–342 (2004).

36. B. D. Griffen, L. Klimes, L. S. Fletcher, N. M. Thometz, Data needs for sea otter bioenergetics modeling. *Conserv. Physiol.* **12**, coae067 (2024).

37. R. T. Paine, Size-limited predation: An observational and experimental approach with the mytilus-pisaster interaction. *Ecology* **57**, 858–873 (1976).

38. Multi-Agency Rocky Intertidal Network (MARINe), Partnership Interdisciplinary Studies Coastal Oceans for of (PISCO), P. Raimondi, MARINe/PISCO: Intertidal: MARINe Coastal Biodiversity Surveys: Point Contact Surveys Summarized (2022); doi: 10.6085/AA/marine_cbs.5.5 .

39. C. P. Winsor, The Gompertz Curve as a Growth Curve. *Proc. Natl. Acad. Sci. U.S.A.* **18**, 1–8 (1932).

40. M. Tim Tinker, P. R. Guimarães Jr., M. Novak, F. M. D. Marquitti, J. L. Bodkin, M. Staedler, G. Bentall, J. A. Estes, Structure and mechanism of diet specialisation: testing models of individual variation in resource use with sea otters. *Ecol. Lett.* **15**, 475–483 (2012).

41. T. A. Dean, J. L. Bodkin, A. K. Fukuyama, S. C. Jewett, D. H. Monson, C. E. O'Clair, G. R. VanBlaricom, Food limitation and the recovery of sea otters following the "Exxon Valdez" oil spill. *Mar. Ecol. Prog. Ser.* **241**, 255–270 (2002).

42. J. A. Estes, M. L. Riedman, M. M. Staedler, M. T. Tinker, B. E. Lyon, Individual variation in prey selection by sea otters: Patterns, causes and implications. *J. Anim. Ecol.* **72**, 144–155 (2003).

43. K. L. Laidre, R. J. Jameson, Foraging patterns and prey selection in an increasing and expanding sea otter population. *J. Mammal.* **87**, 799–807 (2006).

44. C. J. Law, M. T. Tinker, J. A. Fujii, T. Nicholson, M. Staedler, J. A. Tomoleoni, C. Young, R. S. Mehta, Tool use increases mechanical foraging success and tooth health in southern sea otters (Enhydra lutris nereis). *Science* **384**, 798–802 (2024).

45. R. S. Ostfeld, Foraging strategies and prey switching in the California sea otter. *Oecologia* **53**, 170–178 (1982).

46. J. Watt, D. B. Siniff, J. A. Estes, Inter-decadal patterns of population and dietary change in sea otters at Amchitka Island, Alaska. *Oecologia* **124**, 289–298 (2000).

47. B. Carpenter, A. Gelman, M. D. Hoffman, D. Lee, B. Goodrich, M. Betancourt, M. Brubaker, J. Guo, P. Li, A. Riddell, Stan: A probabilistic programming language. *J. Stat. Softw.* **76**, 1–32 (2017).

48. *A language and environment for statistical computing* (R Foundation for Statistical Computing, Vienna, Austria); https://www.R-project.org/.

49. B. B. Hatfield, J. L. Yee, M. C. Kenner, J. A. Tomoleoni, "California sea otter (Enhydra lutris nereis) census results, spring 2019" (1118, U.S. Geological Survey, 2019); https://doi.org/10.3133/ds1118.

50. J. M. Engle, L. Anderson, J. L. Burnaford, M. Douglas, D. P. Lohse, A. Parsons-Field, "Unified Monitoring Protocols for the Multi-Agency Rocky Intertidal Network" (BOEM Cooperative agreement no. M19AC00023, Bureau of Ocean Energy Management, Pacific OCS Region, 2022); https://transfer.natureserve.org/download/USGS-NRMP/RecordSet5_2011-09-15/Proto214_Unified_Monitoring_Protocols_Multi_Agency_Rocky_Intertidal_Network_Photoplot_Protocol.pdf.

51. N. M. Thometz, M. M. Staedler, J. A. Tomoleoni, J. L. Bodkin, G. B. Bentall, M. T. Tinker, Trade-offs between energy maximization and parental care in a central place forager, the sea otter. *Behav. Ecol.* **27**, 1552–1566 (2016).

52. M. T. Tinker, D. F. Doak, J. A. Estes, B. B. Hatfield, M. M. Staedler, J. L. Bodkin, Incorporating diverse data and realistic complexity into demographic estimation procedures for sea otters. *Ecol. Appl.* **16**, 2293–2312 (2006).

53. K. Ralls, B. B. Hatfield, D. B. Siniff, Foraging patterns of California sea otters as indicated by telemetry. *Can. J. Zool.* **73**, 523–531 (1995).
